# Optimal obesity- and lipid-related indices for predicting type 2 diabetes in middle-aged and elderly Chinese

**DOI:** 10.1038/s41598-024-61592-4

**Published:** 2024-05-13

**Authors:** Xiaoyun Zhang, Ying Wang, Yuqing Li, Jiaofeng Gui, Yujin Mei, Xue Yang, Haiyang Liu, Lei-lei Guo, Jinlong Li, Yunxiao Lei, Xiaoping Li, Lu Sun, Liu Yang, Ting Yuan, Congzhi Wang, Dongmei Zhang, Jing Li, Mingming Liu, Ying Hua, Lin Zhang

**Affiliations:** 1 Department of Graduate School, Wannan Medical College, 22 Wenchang West Road, Higher Education Park, Wuhu City, An Hui Province People’s Republic of China; 2 Student Health Center, Wannan Medical College, 22 Wenchang West Road, Higher Education Park, Wuhu City, An Hui Province People’s Republic of China; 3 Department of Surgical Nursing, School of Nursing, Jinzhou Medical University, No. 40, Section 3, Songpo Road, Linghe District, Jinzhou City, Liaoning Province People’s Republic of China; 4 Department of Occupational and Environmental Health, Key Laboratory of Occupational Health and Safety for Coal Industry in Hebei Province, School of Public Health, North China University of Science and Technology, Tangshan, Hebei Province People’s Republic of China; 5 Obstetrics and Gynecology Nursing, School of Nursing, Wannan Medical College, 22 Wenchang West Road, Higher Education Park, Wuhu City, An Hui Province People’s Republic of China; 6 Department of Emergency and Critical Care Nursing, School of Nursing, Wannan Medical College, 22 Wenchang West Road, Higher Education Park, Wuhu City, An Hui Province People’s Republic of China; 7 Department of Internal Medicine Nursing, School of Nursing, Wannan Medical College, 22 Wenchang West Road, Higher Education Park, Wuhu City, An Hui Province People’s Republic of China; 8 Department of Pediatric Nursing, School of Nursing, Wannan Medical College, 22 Wenchang West Road, Higher Education Park, Wuhu City, An Hui Province People’s Republic of China; 9 Department of Surgical Nursing, School of Nursing, Wannan Medical College, 22 Wenchang West Road, Higher Education Park, Wuhu City, An Hui Province People’s Republic of China; 10 Rehabilitation Nursing, School of Nursing, Wannan Medical College, 22 Wenchang West Road, Higher Education Park, Wuhu City, An Hui Province People’s Republic of China

**Keywords:** Type 2 diabetes, Obesity, Anthropometric indices, Cross-sectional study, Middle-aged and elderly Chinese, Diabetes, Dyslipidaemias, Obesity, Diabetes, Obesity, Risk factors, Predictive markers

## Abstract

To investigate the screening and predicting functions of obesity- and lipid-related indices for type 2 diabetes (T2D) in middle-aged and elderly Chinese, as well as the ideal predicted cut-off value. This study's data comes from the 2011 China Health and Retirement Longitudinal Study (CHARLS). A cross-sectional study design was used to investigate the relationship of T2D and 13 obesity- and lipid-related indices, including body mass index (BMI), waist circumference (WC), waist–height ratio (WHtR), visceral adiposity index (VAI), a body shape index (ABSI), body roundness index (BRI), lipid accumulation product (LAP), conicity index (CI), Chinese visceral adiposity index (CVAI), triglyceride- glucose index (TyG index) and its correlation index (TyG-BMI, TyG-WC, TyG-WHtR). The unadjusted and adjusted correlations between 13 indices and T2D were assessed using binary logistic regression analysis. The receiver operating characteristic curve (ROC) was used to determine the usefulness of anthropometric indices for screening for T2D and determining their cut‑off value, sensitivity, specificity, and area under the curve (AUC). The study comprised 9488 people aged 45 years or above in total, of whom 4354 (45.89%) were males and 5134 (54.11%) were females. Among them were 716 male cases of T2D (16.44%) and 870 female cases of T2D (16.95%). A total of 13 obesity- and lipid-related indices were independently associated with T2D risk after adjusted for confounding factors (*P* < 0.05). According to ROC analysis, the TyG index was the best predictor of T2D among males (AUC = 0.780, 95% CI 0.761, 0.799) and females (AUC = 0.782, 95% CI 0.764, 0.799). The AUC values of the 13 indicators were higher than 0.5, indicating that they have predictive values for T2D in middle-aged and elderly Chinese. The 13 obesity- and lipid-related indices can predict the risk of T2D in middle‑aged and elderly Chinese. Among 13 indicators, the TyG index is the best predictor of T2D in both males and females. TyG-WC, TyG-BMI, TyG-WHtR, LAP, and CVAI all outperformed BMI, WC, and WHtR in predicting T2D.

## Introduction

Diabetes is a chronic disease, which has become an observable global public health problem^1^. According to statistics, the global prevalence of diabetes among adults aged 20–79 will be 10.5% (536.6 million people) in 2021 and will rise to 12.2% (783.2 million people) in 2045^2^. Type 2 diabetes (T2D) is the most common form of diabetes, accounting for more than 90% of diabetes cases^3^. Notably, macrovascular problems including coronary heart disease and stroke, as well as microvascular disorders like diabetic kidney disease, retinopathy, and peripheral neuropathy, are common complications of T2D patients^4^. The onset of T2D and the complications that it causes reduce people's quality of life and create significant economic and social burdens^3,5^. Particularly for China, which has the highest prevalence of T2D ^6^. According to the research data of He W et al^7^., the prevalence of T2D in Xiamen is significantly increasing, especially among young people. Similarly, Wang Z et al^8^. also found that the prevalence of T2D in Beijing is gradually increasing, and the prevalence of T2D in women is higher than that in men. Although the prevalence of T2D varies from place to place due to differences in economy, culture, lifestyle, eating habits, and other factors, it is generally on the rise.

Numerous studies have revealed that a wide range of lifestyle variables, such as sedentary behavior, psychological stress, smoking, and being overweight or obese, are extremely important in the development of T2D^9^. Obesity has been proven to increase the risk of T2D by leading to insulin resistance(IR), and lipid metabolism disorders. Obese individuals, due to functional disorders, release a large amount of free fatty acids, reactive oxygen species, and pro-inflammatory cytokines from their adipocytes, which can lead to IR early on and β cell dysfunction^10,11^. IR is a metabolic state in which insulin-dependent tissues become less sensitive to the action of insulin, resulting in ineffective conversion of blood sugar into energy, increasing blood sugar^12^. Long-term IR can lead to a decline in pancreatic islets β cell function and the reduction in insulin secretion may eventually lead to the occurrence of diabetes^13,14^. In addition, obesity can affect lipid metabolism, often accompanied by lipid abnormalities such as hypertriglyceridemia and high-density lipoprotein cholesterol, which further exacerbate IR^15^. When the lipid index increases, more lipid metabolites accumulate in the body, which increases the risk of diabetes, hyperlipidemia, and hyperuricemia^16–18^. As a result, the prevalence of T2D often increased simultaneously with the global rise in obesity prevalence^19^. Some studies^20–22^ have shown that about 90% of patients with diabetes develop T2D, which is mainly related to overweight. With the highest prevalence of overweight and obesity in the world, China may have 789.95 million overweight or obese people by 2030^23^. Given the enormous number of obese people, it is crucial to use simple and efficient methods to identify high-risk populations for T2D, to prevent and treat it as early as possible.

Currently, many studies^24–27^ have shown that efficient and low-cost obesity- and lipid-related indices are associated with IR and T2D. In general, 13 obesity- and lipid-related indices can be used to describe the status of fat accumulation and the amount of lipid metabolites in the body: waist circumference (WC), body mass index (BMI), waist–height ratio (WHtR), visceral adiposity index (VAI), a body shape index (ABSI), body roundness index (BRI), lipid accumulation product (LAP), conicity index (CI), Chinese visceral adiposity index (CVAI), triglyceride-glucose (TyG) index and its correlation index (TyG-BMI, TyG-WC, TyG-WHtR). Mounting studies have suggested to use of these indices to identify high-risk populations for T2D^28–30^. However, there is still controversy over the usefulness of various indices in predicting T2D. This is because anthropometric indices are influenced by factors such as race, age, and gender, and body composition may vary by population and ethnicity^31–33^. Moreover, most studies^33–35^ did not comprehensively compare the predictive ability of obesity- and lipid-related indices for T2D.

Thus, the purpose of this study was to investigate the screening and prediction functions of obesity- and lipid-related indices for the risk of T2D in middle-aged and elderly Chinese, as well as the ideal predicted cut-off value for providing a foundation for T2D prevention and treatment.

## Materials and methods

### Study design and setting

This cross-sectional study followed the ‘Strengthening the Reporting of Observational Studies in Epidemiology’ checklist, we confirm that all methods were performed in accordance with the relevant guidelines and regulations^36^. Data for the current study were obtained from The China Health and Retirement Longitudinal Study (CHARLS), a nationally representative cohort study of China's population of middle-aged and older adults aged from 45 to 101^37^. Without any direct interaction with people, all data are provided in the open as microdata at http://charls.pku.edu.cn/ index/zh-cn.html. Before data collection, all participants provided informed approval, and the study was approved by the Ethics Committee of Peking University's China Center for Economic Research.

### Participants

The individuals for this study were drawn from the China Health and Retirement Longitudinal Study (CHARLS). This study used data from CHARLS Wave1, and participants recruited from May 2011 to March 2012 were included in our study. We excluded individuals who met any of the criteria at baseline (1) type 2 diabetes data missing, (2) one of the 13 indices missing, (3) age/sex/educational levels/marital status/current residence/current smoking/alcohol drinking/taking activities/having regular exercise/ chronic diseases missing. After missing data subjects were excluded, our study included 9,488 aged 45 years and above individuals after missing data subjects were eliminated. A total of 4354 (45.89%) were male and 5134 (54.11%) were female were included.

### Definition of T2D

According to the latest definition of T2D by the International Diabetes Association. Fasting plasma glucose (FPG) levels of 7.0 mmol/L or higher, a 2-h plasma glucose level of 11.1 mmol/L or higher, and a prior self-reported diagnosis of T2D were used to describe the condition. The T2D diagnosis standards were the same as in earlier studies^35,38^.

### Anthropometric indices

Certified medical personnel took anthropometric measures. Body weight and height were estimated to be the closest values of 0.1 kg and 0.5 cm, respectively, using standard medical equipment. After the expiratory breath, WC was measured on both sides between the iliac crest and the lower ribs. BMI was measured with weight (kg) / height^2^ (m^2^). WHtR is defined as WC (cm) / height (cm). The VAI, CVAI, LAP, and TyG-index require TG and high-density lipoprotein cholesterol (HDL-C) to be obtained by invasive examination for calculation. The TyG index is the result of a computation using TG and glucose^39^. Except for WC, the remaining anthropometric indices were calculated using the following Eqs^40–44^:

1 $${\text{BMI}} = \frac{{W{\text{eight}}}}{{H{\text{eight}}^{2} }}$$

2 $${\text{WHtR }} = \frac{WC}{{H{\text{eight}}}}$$

3 $${\text{Males}}:{\text{ VAI}} = \frac{WC}{{39.68 + (1.88 \times BMI)}} \times \frac{TG}{{1.03}} \times \frac{1.31}{{HDL}}$$

$${\text{Females}}:{\text{ VAI}} = \frac{WC}{{36.58 + (1.89 \times BMI)}} \times \frac{TG}{{0.81}} \times \frac{1.52}{{HDL}}$$

4 $${\text{ABSI}} = \frac{WC}{{H{\text{eight}}^{\frac{1}{2}} \times BMI^{\frac{2}{3}} }}$$

5 $${\text{BRI}} = 364.2 - 365.5\sqrt {1 - \left( {\frac{{(WC\left( m \right)/\left( 2\pi \right))^{2} }}{{\left( {0.5 \times Height\left( m \right)} \right)^{2} }}} \right)} { }$$

6 $${\text{Males}}:{\text{ LAP}} = \, \left[ {{\text{WC }}\left( {{\text{cm}}} \right) - {65}} \right] \, \times {\text{TG }}\left( {{\text{mmol}}/{\text{l}}} \right)$$

$${\text{Females}}:{\text{ LAP}} = \, \left[ {{\text{WC }}\left( {{\text{cm}}} \right) - {58}} \right] \, \times {\text{TG }}\left( {{\text{mmol}}/{\text{l}}} \right)$$

7 $${\text{CI}} = \frac{{WC\left( {\text{m}} \right)}}{{0.019\sqrt {\frac{{{\text{weight}}\left( {{\text{kg}}} \right)}}{{{\text{height}}\left( {\text{m}} \right)}}} }}$$

Males:

8 $${\text{CVAI}} = - {267}.{93} + 0.{68} \times {\text{age}} + 0.0{3} \times {\text{BMI }}\left( {{\text{kg}}/{\text{m}^{2}}} \right) \, + {4}.00 \times {\text{WC }}\left( {{\text{cm}}} \right) + {22}.00 \times {\text{Log}}_{{{1}0}} {\text{TG }}\left( {{\text{mmol}}/{\text{l}}} \right) - {16}.{32} \times {\text{HDL}} - {\text{C}}\left( {{\text{mmol}}/{\text{l}}} \right)$$

Females:

$${\text{CVAI}} = - {187}.{32} + {1}.{71} \times {\text{age}} + {4}.{32} \times {\text{BMI }}\left( {{\text{kg}}/{\text{m}^{2}}} \right) \, + {1}.{12} \times {\text{WC }}\left( {{\text{cm}}} \right) + {39}.{76} \times {\text{Log}}_{{{1}0}} {\text{TG }}\left( {{\text{mmol}}/{\text{l}}} \right) - {11}.{66} \times {\text{HDL}} - {\text{C}}\left( {{\text{mmol}}/{\text{l}}} \right)$$

9 $${\text{TyG index}} = {\text{Ln}}\left[ {\left( {{\text{TG}}\left( {{\text{mg}}/{\text{dl}}} \right) \times {\text{glucose}}\left( {{\text{mg}}/{\text{dl}}} \right)/{2}} \right)} \right]$$

10 $${\text{TyG}} - {\text{BMI}} = {\text{ TyG}} \times {\text{BMI}}$$

11 $${\text{TyG}} - {\text{WC}} = {\text{ TyG}} \times {\text{WC}}$$

12 $${\text{TyG }} - {\text{WHtR}} = {\text{ TyG}} \times {\text{WHtR}}$$

### Covariates

Socio-demographic characteristics including age, sex (1 = male, 2 = female), education levels, marital status, current residence, current smoking, alcohol drinking, taking activities, having regular exercise, and chronic disease were collected by the self-reported questionnaire. These categories have been used extensively in our previous research^45–48^.

1. age was sorted into 45–54/55–64/65–74/above 75 years old.
2. education levels were classified into illiterate/less than elementary school/high school/and above vocational school.
3. marital status was classified into single/married;
4. current residence included urban/rural.
5. current smoking was categorized into no smoker/former smoker/current smoker.
6. alcohol drinking was divided into never drinking/ less than once a month/more than once a month.
7. taking activities were sorted into ever (at least once a month) /never.
8. having regular exercise included no exercise/less than exercises/regular exercises.
9. the counts of chronic disease were classified into 0/1–2/ 3–14.

### Statistical analysis

All statistical analysis was performed using IBM SPSS version 25.0 (IBM Corp., Armonk, NY). Categorical variables were expressed as frequency and percent. Chi-square test and one-way ANOVA were performed for the comparison of dichotomous or multiple categorical variables. Means and standard deviations were used to express continuous variables. To evaluate changes in gender average distribution, an independent sample t-test was used. The unadjusted and adjusted correlations between obesity- and lipid-related indicators and T2D were assessed using binary logistic regression analysis. After adjusting for age, education level, marital status, current residence, current smoking, alcohol drinking, taking activities, having regular exercise, and chronic diseases, the odds ratio (OR) and 95% confidence intervals (95%CIs) of each obesity- and lipid-related indices with T2D were calculated. The receiver operating characteristic curve (ROC) was used to calculate the area under the curve (AUC) of each indicator as a predictive factor for T2D. The indicator with the highest AUC was regarded as the most accurate, and the closer the AUC was to 1, the more accurate the prediction would be. Calculations were made for the obesity- and lipid-related markers' sensitivity, specificity, cut-off points, and Youden index [maximum (sensitivity + specificity-1)]. Statistical significance was defined as a *P* < 0.05.

### Ethics approval and consent to participate

All data are openly published as microdata at http://opendata.pku.edu.cn/dataverse/CHARLS with no direct contact with all participants. Approval for this study was given by the medical ethics committee of Wannan Medical College (approval number 2021–3). The patients/participants provided their written informed consent to participate in this study.

## Results

Table 1 shows the baseline characteristics of participants with full samples. The study comprised 9488 people over the age of 45 in total, of whom 4354 (45.89%) were male and 5134 (54.11%) were female. The mean WC, BMI, WHtR, VAI, ABSI, BRI, LAP, CI, CVAI, TyG index, TyG-BMI, TyG-WC, TyG-WHtR, and HbA1c in females are higher than in males (*P* < 0.05). We also observed significant differences in age, education levels, marital status, current smoking, alcohol drinking, and chronic diseases between males and females (*P* < 0.05). However, there was no statistically significant difference in the current residence, taking activities, and having regular exercise between males and females (*P* > 0.05). Because of these significant differences between males and females (*P* < 0.05), we performed the main analyses separately by sex.

**Table 1:** Characteristics of participants with full samples (*N* = 9488).

Variables	Male	Female	Total	t/χ2	*P*
	*N* (%)	*N* (%)	*N* (%)		
N	4354(45.89%)	5134(54.11%)	9488(100%)		
Age(years)				83.541	0.000
45–54	1274(29.26)	1922(37.44)	3196(33.68)		
55–64	1724(39.60)	1930(37.59)	3654(38.51)		
65–74	989(22.71)	903(17.59)	1892(19.94)		
≥ 75	367(8.43)	379(7.38)	746(7.86)		
Education levels				968.940	0.000
Illiterate	597(13.71)	2185(42.56)	2782(29.32)		
Less than elementary school	3195(73.38)	2604(50.72)	5799(61.12)		
High school	360(8.27)	253(4.93)	613(6.46)		
Above vocational school	202(4.64)	92(1.79)	294(3.10)		
Marital status				71.991	0.000
Single	406(9.32)	775(15.10)	1181(12.45)		
Married	3948(90.68)	4359(84.90)	8307(87.55)		
Current residence				0.750	0.387
Rural	4012(92.15)	4755(92.62)	8767(92.40)		
Urban	342(7.85)	379(7.38)	721(7.60)		
Current smoking				4521.237	0.000
No	1075(24.69)	4733(92.19)	5808(61.21)		
Former smoke	734(16.86)	95(1.85)	829(8.74)		
Current smoke	2545(58.45)	306(5.96)	2851(30.05)		
Alcohol drinking				2162.870	0.000
No	1920(44.10)	4516(87.96)	6436(67.83)		
Less than once a month	470(10.79)	255(4.97)	725(7.64)		
More than once a month	1964(45.11)	363(7.07)	2327(24.53)		
Taking activities				0.789	0.374
No	2138(49.10)	2568(50.02)	4706(49.60)		
Yes	2216(50.90)	2566(49.98)	4782(50.40)		
Having regular exercises				1.150	0.563
No exercise	2708(62.20)	3145(61.26)	5853(61.69)		
Less than exercises	814(18.70)	1001(19.50)	1815(19.13)		
Regular exercises	832(19.11)	988(19.24)	1820(19.18)		
Chronic diseases(counts)				20.702	0.000
0	1422(32.66)	1474(28.71)	2896(30.52)		
1–2	2161(49.63)	2623(51.09)	4784(50.42)		
3–14	771(17.71)	1037(20.20)	1808(19.06)		
FPG(mg/dl)	109.99 ± 35.44	109.77 ± 36.90	109.87 ± 36.24	0.297	0.766
HbA1c	5.24 ± 0.77	5.29 ± 0.84	5.27 ± 0.81	− 2.888	0.004
WC	84.96 ± 9.81	85.64 ± 10.16	85.33 ± 10.01	− 3.319	0.001
BMI	22.96 ± 3.64	23.99 ± 4.05	23.51 ± 3.90	− 13.002	0.000
WHtR	0.52 ± 0.06	0.56 ± 0.07	0.54 ± 0.07	− 33.311	0.000
VAI	3.96 ± 4.41	6.07 ± 5.72	5.10 ± 5.26	− 20.256	0.000
ABSI	0.08 ± 0.01	0.08 ± 0.01	0.08 ± 0.01	− 10.650	0.000
BRI	3.78 ± 1.14	4.66 ± 1.46	4.26 ± 1.39	− 33.183	0.000
LAP	30.87 ± 33.31	43.74 ± 35.23	37.83 ± 34.95	− 18.262	0.000
CI	1.27 ± 0.08	1.30 ± 0.10	1.29 ± 0.09	− 15.487	0.000
CVAI	95.98 ± 47.50	107.11 ± 43.43	102.00 ± 45.68	− 11.826	0.000
TyG index	8.62 ± 0.66	8.72 ± 0.63	8.68 ± 0.65	− 7.594	0.000
TyG-BMI	198.67 ± 39.64	209.77 ± 41.67	204.68 ± 41.12	− 13.285	0.000
TyG-WC	7.35 ± 1.18	7.49 ± 1.16	7.42 ± 1.17	− 5.840	0.000
TyG -WHtR	4.48 ± 0.69	4.91 ± 0.76	4.71 ± 0.76	− 28.240	0.000

FPG: fasting plasma glucose; HbA1c: glycated hemoglobin; WC: waist circumference; BMI: body mass index; WHtR: waist to height ratio; VAI: visceral adiposity index; ABSI: A body shape index; BRI: body roundness index; LAP: lipid accumulation product; CVAI: Chinese visceral adiposity index; CI: conicity index; TyG index: triglyceride-glucose index; TyG-BMI: TyG related to BMI; TyG-WC: TyG related to WC; TyG-WHtR: TyG related to WHtR.

Table 2 shows the baseline characteristics of the study participants with and without T2D by sex. The cross-sectional analysis included 716 males with T2D (16.44%) and 870 females with T2D (16.95%). Males with T2D had significant differences in age, current smoking, chronic diseases, FPG, HbA1c, WC, BMI, WHtR, VAI, ABSI, BRI, LAP, CI, CVAI, TyG index, TyG-BMI, TyG-WC, and TyG-WHtR (*P* < 0.05). Females with T2D had significant differences in age, current residence, alcohol drinking, taking activities, chronic diseases, FPG, HbA1c, WC, BMI, WHtR, VAI, ABSI, BRI, LAP, CI, CVAI, TyG index, TyG-BMI, TyG-WC, and TyG-WHtR (*P* < 0.05).

**Table 2:** Baseline characteristics of the study participants with and without T2D by sex.

Variables	Male		*χ*^*2*^	*P*	Female		*χ*^*2*^	*P*
	With T2D	Without T2D			With T2D	Without T2D		
	N (%)	N (%)			N (%)	N (%)		
N	716(16.44)	3638(83.56)			870(16.95)	4264(83.05)		
Age(years)			15.064	0.002			37.063	0.000
45–54	167(23.32)	1107(30.43)			249(28.62)	1673(39.24)		
55–64	304(42.46)	1420(39.03)			368(42.30)	1562(36.63)		
65–74	175(24.44)	814(22.37)			187(21.49)	716(16.79)		
≥ 75	70(9.78)	297(8.16)			66(7.59)	313(7.34)		
Education levels			2.262	0.520			2.083	0.555
Illiterate	96(13.41)	501(13.77)			378(43.45)	1807(42.38)		
Less than elementary school	526(73.46)	2669(73.36)			431(49.54)	2173(50.96)		
High school	54(7.54)	306(8.41)			41(4.71)	212(4.97)		
Above vocational school	40(5.59)	162(4.45)			20(2.30)	72(1.69)		
Marital status			0.099	0.753			0.019	0.890
Single	69(9.64)	337(9.26)			130(14.94)	645(15.13)		
Married	647(90.36)	3301(90.74)			740(85.06)	3619(84.87)		
Current residence			2.673	0.102			7.135	0.008
Rural	649(90.64)	3363(92.44)			787(90.46)	3968(93.06)		
Urban	67(9.36)	275(7.56)			83(9.54)	296(6.94)		
Current smoking			11.336	0.003			0.724	0.696
No	182(25.42)	893(24.55)			796(91.49)	3937(92.33)		
Former smoke	149(20.81)	585(16.08)			18(2.07)	77(1.81)		
Current smoke	385(53.77)	2160(59.37)			56(6.44)	250(5.86)		
Alcohol drinking			2.224	0.329			6.967	0.031
No	328(45.81)	1592(43.76)			786(90.34)	3730(87.48)		
Less than once a month	67(9.36)	403(11.08)			40(4.60)	215(5.04)		
More than once a month	321(44.83)	1643(45.16)			44(5.06)	319(7.48)		
Taking activities			1.423	0.233			4.393	0.036
No	337(47.07)	1801(49.51)			407(46.78)	2161(50.68)		
Yes	379(52.93)	1837(50.49)			463(53.22)	2103(49.32)		
Having regular exercises			2.369	0.306			1.596	0.450
No exercise	463(64.66)	2245(61.71)			549(63.10)	2596(60.88)		
Less than exercises	128(17.88)	686(18.86)			159(18.28)	842(19.75)		
Regular exercises	125(17.46)	707(19.43)			162(18.62)	826(19.37)		
Chronic diseases(counts)			114.524	0.000			196.794	0.000
0	155(21.65)	1267(34.83)			152(17.47)	1322(31.00)		
1–2	341(47.63)	1820(50.03)			397(45.63)	2226(52.20)		
3–14	220(30.73)	551(15.15)			321(36.90)	716(16.79)		
FPG(mg/dl)	161.05 ± 61.26	99.94 ± 12.32	− 26.586	0.000	157.79 ± 67.82	99.97 ± 11.62	− 26.070	0.000
HbA1c	5.97 ± 1.44	5.10 ± 0.40	− 16.192	0.000	6.20 ± 1.56	5.10 ± 0.39	− 20.477	0.000
WC	88.72 ± 10.53	84.22 ± 9.49	− 10.616	0.000	88.85 ± 10.10	84.99 ± 10.05	− 10.325	0.000
BMI	24.17 ± 4.18	22.72 ± 3.48	− 8.692	0.000	24.99 ± 4.48	23.78 ± 3.92	− 7.378	0.000
WHtR	0.54 ± 0.06	0.51 ± 0.06	− 11.286	0.000	0.58 ± 0.07	0.56 ± 0.07	− 10.617	0.000
VAI	6.33 ± 7.38	3.49 ± 3.35	− 10.087	0.000	8.99 ± 8.44	5.47 ± 4.77	− 11.905	0.000
ABSI	0.08 ± 0.01	0.08 ± 0.01	− 4.701	0.000	0.08 ± 0.01	0.08 ± 0.01	− 4.655	0.000
BRI	4.24 ± 1.23	3.69 ± 1.10	− 11.088	0.000	5.14 ± 1.57	4.57 ± 1.41	− 10.584	0.000
LAP	50.25 ± 51.98	27.05 ± 26.61	− 11.643	0.000	62.89 ± 47.06	39.83 ± 30.87	− 13.859	0.000
CI	1.30 ± 0.08	1.27 ± 0.08	− 8.380	0.000	1.32 ± 0.09	1.30 ± 0.10	− 7.696	0.000
CVAI	118.90 ± 51.88	91.47 ± 45.25	− 13.196	0.000	129.02 ± 43.40	102.64 ± 42.06	− 16.770	0.000
TyG index	9.24 ± 0.78	8.50 ± 0.56	− 24.176	0.000	9.30 ± 0.72	8.60 ± 0.54	− 27.093	0.000
TyG-BMI	224.27 ± 48.84	193.63 ± 35.46	− 15.976	0.000	232.67 ± 46.17	205.10 ± 39.08	− 16.454	0.000
TyG-WC	821.92 ± 134.91	717.41 ± 106.25	− 19.570	0.000	827.60 ± 121.56	732.57 ± 108.26	− 21.392	0.000
TyG-WHtR	5.01 ± 0.78	4.38 ± 0.62	− 20.603	0.000	5.43 ± 0.80	4.80 ± 0.71	− 21.569	0.000

FPG: fasting plasma glucose; HbA1c: glycated hemoglobin; WC: waist circumference; BMI: body mass index; WHtR: waist to height ratio; VAI: visceral adiposity index; ABSI: A body shape index; BRI: body roundness index; LAP: lipid accumulation product; CVAI: Chinese visceral adiposity index; CI: conicity index; TyG index: triglyceride-glucose index; TyG-BMI: TyG related to BMI; TyG-WC: TyG related to WC; TyG-WHtR: TyG related to WHtR.

**Figure 1:**
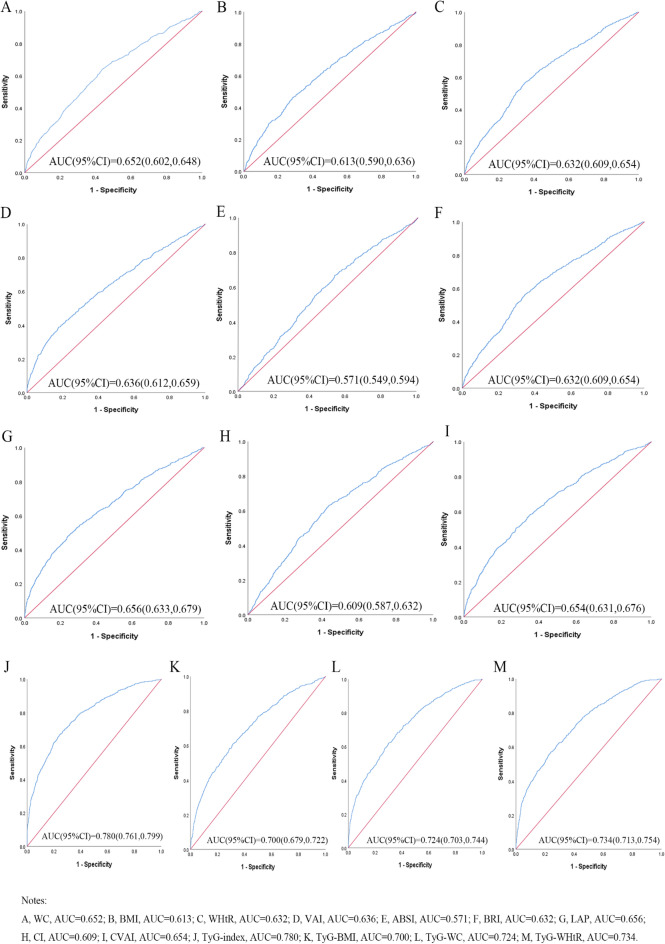
The ROC curves of each indicator in the prediction of T2D risk in males.

**Figure 2:**
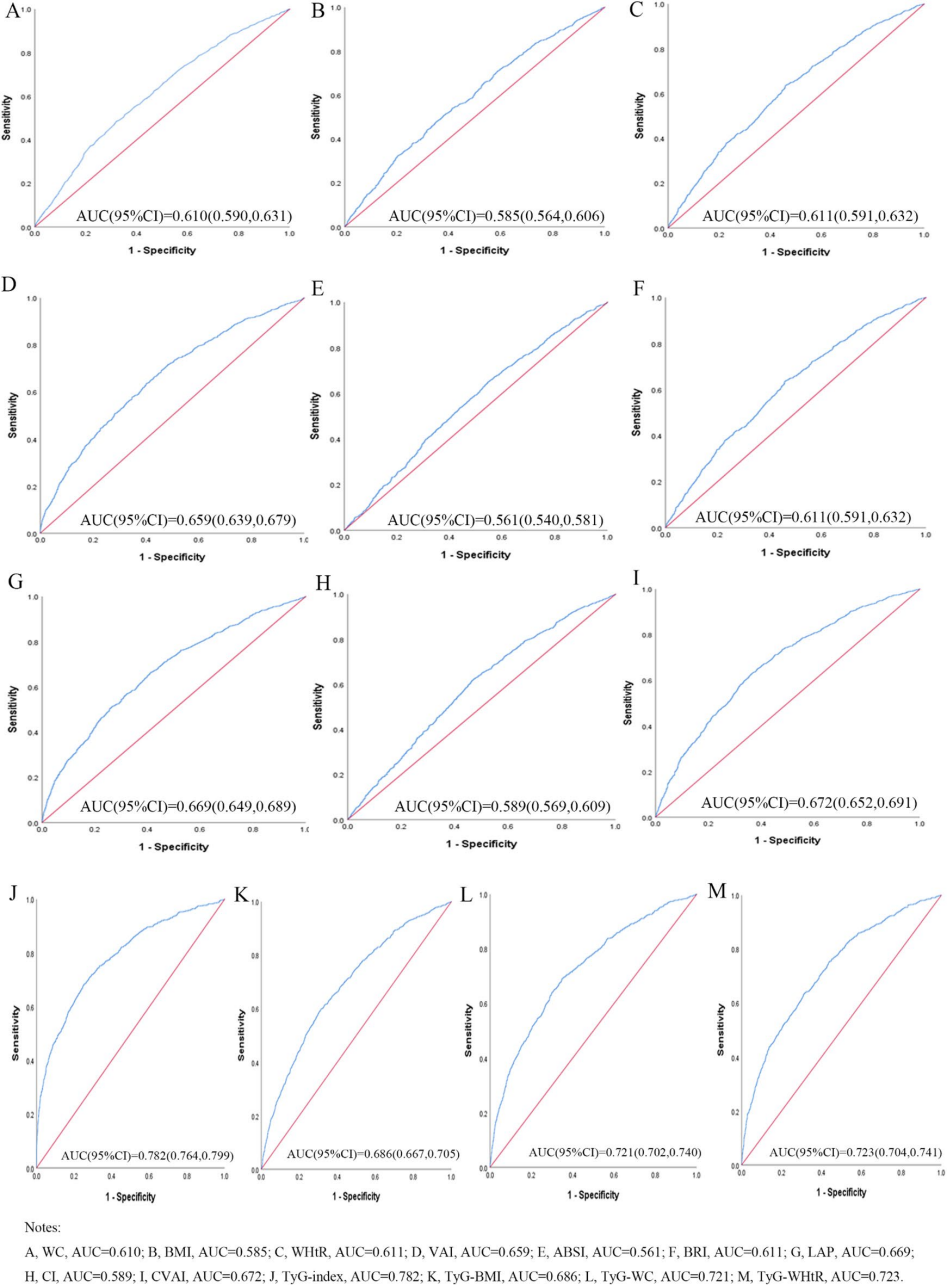
The ROC curves of each indicator in the prediction of T2D risk in females.

Table 3 shows the cut-off value between the area under the curve, sensitivity, and specificity for obesity- and lipid-related indices to detect T2D by sex. The ROC curves of each indicator in the prediction of T2D risk in males and females are shown in Figs. 1 and 2, respectively. As shown in the table and figures, among males, the TyG index was the best predictor of T2D (AUC = 0.780, 95% CI: 0.761, 0.799, and optimal cutoffs = 8.838). Meanwhile, TyG-WHtR (AUC = 0.734, 95%CI: 0.713, 0.754, and optimal cutoffs = 4.716) had similar predictive values. Moreover, among females, the TyG index was the most accurate predictor of T2D (AUC = 0.782, 95%CI: 0.764, 0.799, and optimal cutoffs = 8.940), followed by TyG-WHtR (AUC = 0.723, 95%CI: 0.704, 0.741, and optimal cutoffs = 5.131), and TyG-WC (AUC = 0.721, 95%CI: 0.702, 0.740, and optimal cutoffs = 769.129). All of the above indicators have statistical significance (*P* < 0.05). From the overall data, the AUC values of the above 13 indicators were higher than 0.5, indicating that they have predictive values for T2D in middle-aged and elderly Chinese.

**Table 3:** Cut‑off between area under the curve, sensitivity and specificity for obesity‑ and lipid‑related indices to detect T2D by sex.

	WC	BMI	WHtR	VAI	ABSI	BRI	LAP	CI	CVAI	TyG index	TyG-BMI	TyG-WC	TyG -WHtR
Male													
Area under curve	0.625	0.613	0.632	0.636	0.571	0.632	0.656	0.609	0.654	0.780	0.700	0.724	0.734
Std. Error	0.012	0.012	0.011	0.012	0.012	0.011	0.012	0.012	0.011	0.010	0.011	0.011	0.010
95%CI	0.602,0.648	0.590,0.636	0.609,0.654	0.612,0.659	0.549,0.594	0.609,0.654	0.633,0.679	0.587,0.632	0.631,0.676	0.761,0.799	0.679,0.722	0.703,0.744	0.713,0.754
*P*-value	0.000	0.000	0.000	0.000	0.000	0.000	0.000	0.000	0.000	0.000	0.000	0.000	0.000
Optimal cutoffs	84.650	24.238	0.532	4.881	0.082	3.971	33.755	1.278	106.900	8.838	206.505	767.931	4.716
J-Youden	0.202	0.179	0.219	0.211	0.130	0.219	0.240	0.195	0.231	0.426	0.296	0.323	0.331
Sensitivity (%)	64.2%	46.5%	56.4%	40.1%	67.0%	56.4%	49.6%	63.1%	58.1%	66.8%	61.0%	62.2%	61.5%
Specificity (%)	56.0%	71.4%	65.5%	81.0%	46.0%	65.5%	74.4%	56.4%	65.0%	75.8%	68.6%	70.1%	71.6%
( +) Likelihood ratio	1.459	1.626	1.635	2.111	1.241	1.635	1.938	1.447	1.660	2.760	1.943	2.080	2.165
( −) Likelihood ratio	0.639	0.749	0.666	0.740	0.717	0.666	0.677	0.654	0.645	0.438	0.569	0.539	0.538
Female													
Area under curve	0.610	0.585	0.611	0.659	0.561	0.611	0.669	0.589	0.672	0.782	0.686	0.721	0.723
Std. Error	0.010	0.011	0.010	0.010	0.010	0.010	0.010	0.010	0.010	0.009	0.010	0.010	0.010
95%CI	0.590,0.631	0.564,0.606	0.591,0.632	0.639,0.679	0.540,0.581	0.591,0.632	0.649,0.689	0.569,0.609	0.652,0.691	0.764,0.799	0.667,0.705	0.702,0.740	0.704,0.741
*P*-value	0.000	0.000	0.000	0.000	0.000	0.000	0.000	0.000	0.000	0.000	0.000	0.000	0.000
Optimal cutoffs	89.350	24.599	0.561	4.810	0.082	4.571	36.918	1.301	115.018	8.940	221.078	769.129	5.131
J-Youden	0.165	0.127	0.178	0.235	0.106	0.178	0.253	0.152	0.266	0.432	0.285	0.343	0.323
Sensitivity (%)	48.5%	50.8%	63.7%	63.7%	65.2%	63.7%	66.3%	62.4%	63.2%	68.5%	59.0%	69.2%	63.4%
Specificity (%)	68.0%	61.9%	54.1%	59.8%	45.4%	54.1%	59.0%	52.8%	63.4%	74.7%	69.5%	65.1%	68.9%
( +) Likelihood ratio	1.516	1.333	1.388	1.585	1.194	1.388	1.617	1.322	1.727	2.708	1.934	1.983	2.039
( −) Likelihood ratio	0.757	0.795	0.671	0.607	0.767	0.671	0.571	0.712	0.580	0.422	0.590	0.473	0.531

WC: waist circumference; BMI: body mass index; WHtR: waist to height ratio; VAI: visceral adiposity index; ABSI: A body shape index; BRI: body roundness index; LAP: lipid accumulation product; CVAI: Chinese visceral adiposity index; CI: conicity index; TyG index: triglyceride-glucose index; TyG-BMI: TyG related to BMI; TyG-WC: TyG related to WC; TyG-WHtR: TyG related to WHtR.

Table 4 shows the odds ratios (ORs) and 95% confidence interval (CIs) for the associations of obesity- and lipid-related indices with T2D. According to the optimal cutoffs in Table 3, 13 obesity- and lipid-related indices were converted into two-category variables in this investigation. Table 4 is based on the transformed variables. A larger OR, in general, suggests a higher risk factor. Both before and after adjusting for age, educational levels, marital status, current residence, current smoking, alcohol drinking, taking activities, having regular exercise, and chronic disease counts, the odds of the risk of T2D increased progressively with increasing units of obesity- and lipid-related indices for both males and females. After adjusting for all covariates, each unit rises in TyG index, for example, was related to a 6.643-fold (95% CI: 5.547, 7.954) increase in the likelihood of developing T2D in males and 5.957-fold (95% CI: 5.068, 7.002) in females. Each unit increase in TyG-WHtR was linked to a 3.886-fold (95% CI: 3.270, 4.618) increase in the likelihood of developing T2D in males and 3.314-fold (95% CI: 2.833, 3.877) in females. Among the 13 indicators, the correlation between ABSI and T2D was weakest in males (OR = 1.516, 95% CI: 1.278, 1.797) and females (OR = 1.376, 95% CI: 1.163, 1.682). All indices had statistical significance after adjustment of confounding factors (*P* < 0.05).

**Table 4:** Odds ratios (ORs) and 95% confidence interval (CIs) for the associations of obesity- and lipid-related indices with T2D.

Variables	Male				Female			
	Model 1		Model 2		Model 1		Model 2	
	OR (95%CI)	*P*	OR (95%CI)	*P*	OR (95%CI)	*P*	OR (95%CI)	*P*
WC	2.284(1.934,2.697)	0.000	2.205(1.855,2.622)	0.000	1.998(1.724,2.316)	0.000	1.703(1.462,1.983)	0.000
BMI	2.166(1.839,2.551)	0.000	2.094(1.758,2.494)	0.000	1.675(1.447,1.940)	0.000	1.522(1.307,1.773)	0.000
WHtR	2.446(2.079,2.878)	0.000	2.251(1.903,2.662)	0.000	2.058(1.771,2.393)	0.000	1.760(1.506,2.057)	0.000
VAI	2.843(2.397,3.373)	0.000	2.868(2.403,3.423)	0.000	2.603(2.238,3.028)	0.000	2.337(2.002,2.728)	0.000
ABSI	1.608(1.363,1.896)	0.000	1.516(1.278,1.797)	0.000	1.527(1.308,1.782)	0.000	1.376(1.163,1.682)	0.000
BRI	2.453(2.084,2.886)	0.000	2.255(1.907,2.668)	0.000	2.063(1.774,2.398)	0.000	1.764(1.509,2.062)	0.000
LAP	2.851(2.419,3.360)	0.000	2.844(2.394,3.378)	0.000	2.818(2.418,3.285)	0.000	2.476(2.117,2.896)	0.000
CI	2.203(1.867,2.599)	0.000	2.024(1.709,2.397)	0.000	1.848(1.591,2.146)	0.000	1.619(1.379,1.900)	0.000
CVAI	2.570(2.183,3.026)	0.000	2.409(2.034,2.854)	0.000	2.973(2.556,3.459)	0.000	2.516(2.143,2.953)	0.000
TyG index	6.266(5.271,7.450)	0.000	6.643(5.547,7.954)	0.000	6.421(5.478,7.526)	0.000	5.957(5.068,7.002)	0.000
TyG-BMI	3.415(2.893,4.030)	0.000	3.508(2.943,4.182)	0.000	3.269(2.814,3.798)	0.000	2.978(2.549,3.479)	0.000
TyG-WC	3.844(3.253,4.541)	0.000	3.851(3.235,4.585)	0.000	4.182(3.574,4.893)	0.000	3.677(3.132,4.316)	0.000
TyG-WHtR	4.009(3.393,4.737)	0.000	3.886(3.270,4.618)	0.000	3.829(3.288,4.460)	0.000	3.314(2.833,3.877)	0.000

WC: waist circumference; BMI: body mass index; WHtR: waist to height ratio; VAI: visceral adiposity index; ABSI: A body shape index; BRI: body roundness index; LAP: lipid accumulation product; CVAI: Chinese visceral adiposity index; CI: conicity index; TyG index: triglyceride-glucose index; TyG-BMI: TyG related to BMI; TyG-WC: TyG related to WC; TyG-WHtR: TyG related to WHtR.

Model l: unadjusted.

Model 2: adjusting for age, educational levels, marital status, current residence, current smoking, alcohol drinking, taking activities, having regular exercises, and chronic diseases.

## Discussion

In our study, we confirmed the correlation between 13 obesity- and lipid-related indicators and T2D, and compared the predictive ability of these indicators for the risk of T2D. Our study found that participants with T2D were higher than those without T2D in all indicator levels, and the higher the indicator level, the greater the risk of T2D. In addition, through ROC analysis, we found that among the 13 indicators, the TyG index performed the best, followed by TyG-WHtR, TyG-WC, and TyG-BMI.

Our research is consistent with the findings of Ahn N et al., who suggest using the TyG index as a reliable, convenient, and economical diagnostic measurement for the early identification of diabetes in the general population^49^. Previous research has shown that dyslipidemia, which is marked by elevated TG, and IR, is very common among individuals with T2D^50,51^. TyG index which is created through the multiplication of TG by glucose, has shown promise as substitute indices for IR because of their practical and widespread availability^52,53^. TyG-BMI, TyG-WC, and TyG-WHtR are obtained by multiplying TyG with BMI, WC, and WHtR. Several studies suggested that the indices had greater efficacy than the TyG index alone in predicting T2D^31,54^. In our study, the TyG index is the most effective indicator for identifying T2D (AUC = 0.780 in males and 0.782 in females) among all indicators, followed by TyG-WHtR (AUC = 0.734 in males and 0.723 in females), TyG-WC (AUC = 0.724 in males and 0.721 in females), and TyG-BMI (AUC = 0.700 in males and 0.686 in females).

It is worth noting that in addition to the TyG index and TyG-related factors, new visceral obesity indicators LAP and CVAI have also been reported as important predictors of T2D^13,55^. LAP is a lipid accumulation index calculated based on WC and TG, which performs best in predicting metabolic syndrome^56^ and it was superior to BMI in identifying T2D risk^57^. In this study, LAP is second only to TyG-related indices in males (AUC = 0.656, 95% CI: 0.633, 0.679, and optimal cutoff value = 33.755) and females (AUC = 0.669, 95% CI: 0.649, 0.689, and optimal cutoff value = 36.918). In addition, the CVAI calculated based on age, BMI, WC, TG, and HDL-C represents a novel indicator of visceral adipose tissue^58,59^. As described by Han et al., CVAI (AUC = 0.695 in men and 0.707 in women) performs better in predicting the risk of T2D compared to other visceral obesity indices (such as VAI, WC, and BMI)^34^. Similarly, Wei et al. also found that CVAI outperformed WC, BMI, and ABSI in diabetes screening among Chinese adults^60^. In this study, for males, the AUC of CVAI (AUC = 0.654, 95% CI: 0.631, 0.679, and optimal cutoff value = 106.900) was the largest except for the TyG index and its related indices, and LAP. For females, the AUC of CVAI (AUC = 0.672, 95% CI: 0.652, 0.691, and optimal cutoff value = 115.018) was also second only to the TyG index and its related indices. Compared with males, we found that CVAI has a better predictive ability for T2D in females. This interesting result was also found in Tsou MT et al.*’*s study, which may be related to physiological differences in visceral fat deposition and distribution between the genders^61^.

In this study, 13 obesity- and lipid-related indices were converted into two-category variables according to the optimal cutoff point in Table 3. Table 4 is based on the transformed variables. In general, a higher OR indicates a greater risk factor. In Table 4, the OR value of ABSI is much lower than that of the remaining 12 indices in both sexes (OR = 1.516 in males and 1.376 in females), after adjusting for all confounding factors. According to ROC analysis, the AUC value of ABSI is lower than that of the remaining 12 indices in both sexes (AUC = 0.571 in males and 0.561 in females). Consistent with our research results, Chang et al. also found that in terms of predicting the existence of diabetes among the rural population in Northeast China, compared with BRI, ABSI has the weakest predictive ability^30^.

Our research has several limitations. As this study is a cross-sectional study, it is not possible to make causal inferences about the relationship between the 13 indicators and the risk of T2D. Furthermore, the participants in this study are only Chinese aged 45 and above, therefore, this conclusion will limit the generalizability of the results to other countries and ethnic groups.

Additionally, our study also has several advantages. We examined the effects of 13 obesity- and lipid-related indicators on T2D and comprehensively compared their predictive ability for T2D, which was useful for early screening. Second, most of the indicators in this study were simple indicators with strong practicality for use in normal clinical practice to predict T2D. Finally, This study also included 9488 participants who were 45 years of age or older, the large sample size enhanced the generalizability and effectiveness of the research results.

## Conclusion

In this study, 13 obesity- and lipid-related indices were able to significantly predict T2D in middle-aged and elderly Chinese. Among 13 indicators, the TyG index is the best indicator to predict T2D in males and females. At the same time, CVAI, LAP, TyG-BMI, TyG-WC, and TyG-WHtR performed better than traditional indicators (such as BMI, WC, and WHtR) in predicting T2D. It is worth noting that these indicators can serve as simple indicators for predicting T2D in clinical practice and epidemiological investigations, they can provide certain reference values for early identification and prevention of T2D.

## Data Availability

Data can be accessed via http://opendata.pku.edu.cn/dataverse/CHARLS.

## Abbreviations

CHARLS: China Health and Retirement Longitudinal Study
WC: Waist circumference
BMI: Body mass index
WHtR: Waist–height ratio
VAI: Visceral adiposity index
ABSI: A body shape index
BRI: Body roundness index
LAP: Lipid accumulation product
CI: Conicity index
CVAI: Chinese visceral adiposity index
TyG index: Triglyceride-glucose index
TyG-BMI: Triglyceride-glucose related to BMI
TyG-WC: Triglyceride-glucose related to WC
TyG-WHtR: Triglyceride-glucose related to WHtR
T2D: Type 2 diabetes
IR: Insulin resistance
TG: Triglyceride
HDL-C: High-density lipoprotein cholesterol
FPG: Fasting plasma glucose
HbA1c: Glycated hemoglobin
SPSS: Statistical product service solutions
ROC: Receiver operating characteristic curve
AUC: Area under the curve
ORs: Odds ratios
95% CIs: 95% Confidence intervals
